# The Mechanics of Metastasis: Insights from a Computational Model

**DOI:** 10.1371/journal.pone.0044281

**Published:** 2012-09-28

**Authors:** G. Wayne Brodland, Jim H. Veldhuis

**Affiliations:** Department of Civil and Environmental Engineering, University of Waterloo, Waterloo, Ontario, Canada; Institute for Medical Biomathematics, Israel

## Abstract

Although it may seem obvious that mechanical forces are required to drive metastatic cell movements, understanding of the mechanical aspects of metastasis has lagged far behind genetic and biochemical knowledge. The goal of this study is to learn about the mechanics of metastasis using a cell-based finite element model that proved useful for advancing knowledge about the forces that drive embryonic cell and tissue movements. Metastasis, the predominant cause of cancer-related deaths, involves a series of mechanical events in which one or more cells dissociate from a primary tumour, migrate through normal tissue, traverse in and out of a multi-layer circulatory system vessel and resettle. The present work focuses on the dissemination steps, from dissociation to circulation. The model shows that certain surface tension relationships must be satisfied for cancerous cells to dissociate from a primary tumour and that these equations are analogous to those that govern dissociation of embryonic cells. For a dissociated cell to then migrate by invadopodium extension and contraction and exhibit the shapes seen in experiments, the invadopodium must generate a contraction equal to approximately twice that produced by the interfacial tension associated with surrounding cells. Intravasation through the wall of a vessel is governed by relationships akin to those in the previous two steps, while release from the vessel wall is governed by equations that involve surface and interfacial tensions. The model raises a number of potential research questions. It also identifies how specific mechanical properties and the sub-cellular structural components that give rise to them might be changed so as to thwart particular metastatic steps and thereby block the spread of cancer.

## Introduction

**Figure 1:**
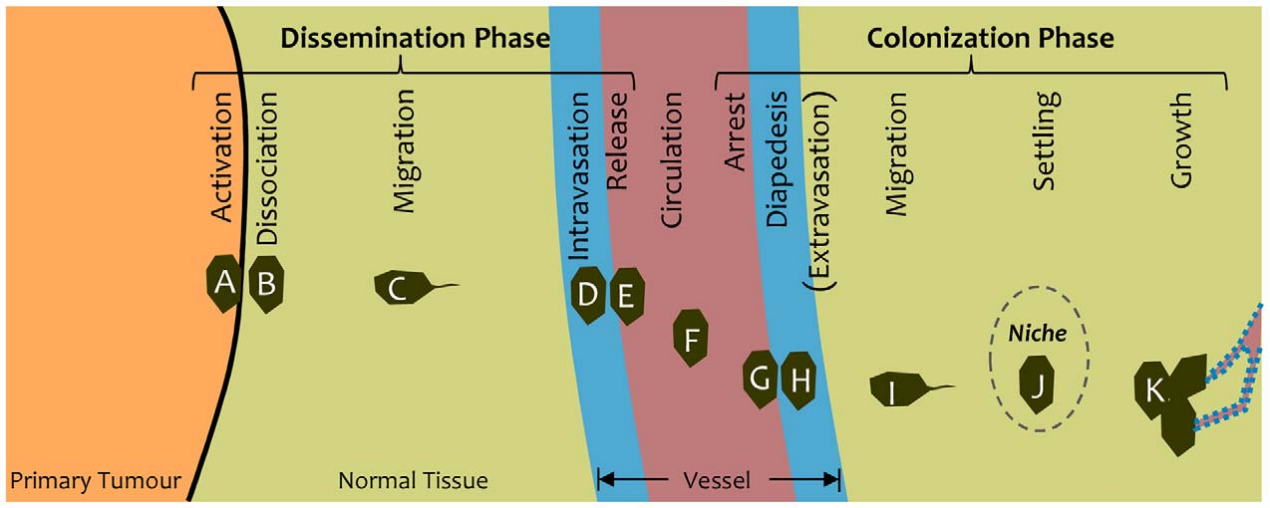
The steps in cancer metastasis. The steps in the metastatic process can be grouped into two phases: dissemination and colonization. For a cancer cell (A) to be disseminated into the body through the blood stream, it must dissociate (B) from a primary tumour, migrate (C) to a blood (or lymph) vessel, enter and traverse the vessel wall (D) and be released (E) into the blood flow. It can then be carried (F) to a different part of the body. Colonization at that new location requires that it arrest on the vessel wall (G), pass through the wall (H), migrate (I) to a suitable niche (dashed ellipse) where it settles (J) and grows into a secondary tumour (K), receiving nutrients through new vessels formed by angiogenesis (dotted outline). This article focuses on the dissemination phase.

Although most cancer-related deaths are a consequence of metastases, the forces that drive its characteristic cell motions are still poorly understood and, as an unfortunate result, a general framework for devising prevention strategies does not exist [1]–[3]. Efforts to find such a framework have been confounded by the wide variety of distinct cancer types that occur, the heterogeneous nature of individual cancer tumours, the complexity of the metastasis process (Fig. 1) and the fact that the cell motions of interest typically occur deep inside the body where direct observation is difficult if not impossible [1], [4]–[6]. Although much attention has been devoted to the genetic and biochemical aspects of metastases [1], [2], [6], [7], interest in its mechanical aspects is relatively new [3], [8]–[11].

More than a decade ago, the field of developmental biology saw a renewed interest in the mechanics of cells and tissues, and this led to dramatic advances in our understanding of how cells and tissues move during embryogenesis. Mechanics provided the missing bridge between newly-acquired genetic and biochemical knowledge on the one hand and observations of tissue movements and associated phenotypes on the other, and computational models were a central component of this advance [12]–[15]. Models played an important role because they made it possible to pose and test hypotheses that could not be tested *in vivo* or *in vitro*. For example, they allowed mechanical properties to be adjusted quantitatively and incrementally, either arbitrarily or based on assumed genetic changes [16]–[18]. Models allowed animal-to-animal variability to be circumvented since identical starting configurations could be used for a whole series of *in silico* experiments, if desired. They also made it possible to better interpret experiments [18], [19] and to trace in detail the causal sequences through which genetic and other system modifications affect these motions and the medical outcomes they ultimately produce [17], [18], [20]. These models showed that the forces at work in seemingly simple motions such as cell sorting or the rolling up of a sheet of tissue into a tube during neurulation were often counterintuitive, and that suitable models based on principles of physics are a necessary complement to experiments if one seeks to reliably test ideas about the forces that drive particular cell and tissue motions [15], [17], [21]–[24]. These models and their associated experiments also showed that movements of cells and tissues in embryos can be driven by a variety of mechanisms including interfacial and surface tensions and cellular protrusions.

Two fundamental ideas came out of this work. The first was an explicit understanding that cells can only move when they are acted on by mechanical forces. In a sense, it does not matter what genes are expressed, what signalling molecules are present, what chemotactic gradients are generated, what pathways are activated or what structural components are present. If they do not work together so as to generate the required mechanical forces, no movement will take place. This argument does not reduce the importance of genetic, biochemical and ultrastructural analyses in understanding cell movement, but it highlights the important fact that if these factors do not produce suitable mechanical forces, cells will not move. The second fundamental idea was that for a cell to move, it must be acted on by forces that are not in balance (i.e., that are out of equilibrium) with each other. Thus, if a cell is subjected to equal tensile forces in all directions, or forces that sum to zero (in a vector sense), it will not move. Movement requires that the forces acting on that cell be out of balance. That is to say, there must be differential forces. Furthermore, these studies showed that the motions can depend on details of the acting forces, and that changes in the driving forces can change medical outcomes [17], [25].

**Figure 2:**
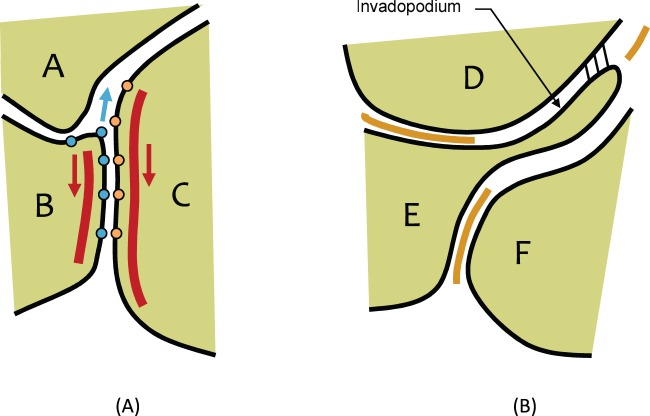
Cell actions that produce net boundary (interfacial) tensions. (A) Contraction of microfilaments (red curves) and cell membranes (black curves) tends to cause the interface between cells B and C to shorten, while cell-cell adhesion systems (orange and blue dots) tend to make it elongate. The result of the joint action of these and other sub-cellular structural components is a net interfacial tension γ_BC_ (Fig. 3B). (B) Invadopodia (as from cell E) can push through where the extra-cellular matrix (orange curves) has been dissolved. If attachments are made to neighbouring cells (short black lines) and the invadopodium contracts like a lamellipodium, the tension γ_DF_ along the interface between cells D and F becomes elevated.

Obvious parallels exist between morphogenetic movements of cells in embryos and metastasizing cancer cells [26] and the concept of using models originally designed to study embryogenesis to investigate cancer cell motions is appealing. Both situations involve canonical patterns of movement driven by cytoskeletal components, cell adhesion systems, surface tensions and lamellipodia (Fig. 2); the cell and tissue geometries and types are similar; and so are at least some of the governing genes [1], [17], [18], [27], [28]. One might wonder whether mechanics-based computational models could bring insights to the study of metastasis, and one goal of this study is to answer that question.

**Figure 3:**
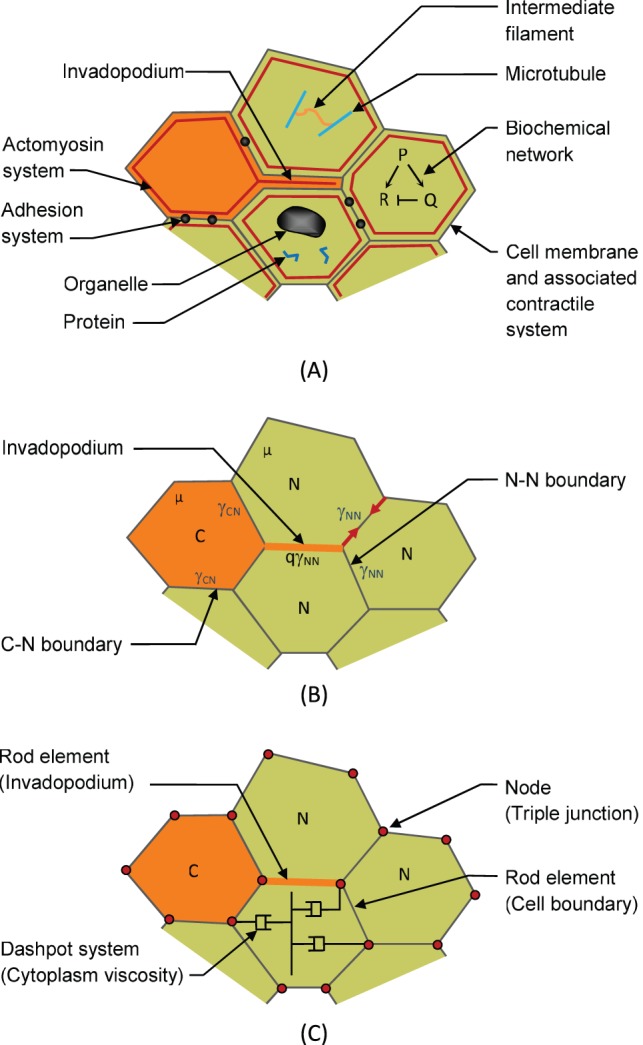
Construction of the finite element model. (A) Biochemical networks regulate the construction of signaling and structural proteins and lead to assembly and regulation of mechanical components. These mechanical components (especially actomyosin and adhesion systems) generate net interfacial tensions γ along the cell edges [41], [42] (B). Cancer cells (type “C”) generate invadopodia which push their way between the normal cells (labelled as type “N”). They then contract with a force assumed to be q times the tension γ_NN_ that acts along the boundaries of the surrounding cells. In the finite element model, the edge forces are generated using rods that lie along each edge and that have zero stiffness but carry a constant tension γ that is specific to the cell types that form the boundary. The viscosity µ of the contents of the cells is modeled using a series of orthogonal dashpots [46], only one set of which is illustrated in the figure.

The process of metastasis involves a series of steps (Fig. 1) that can be divided into two phases. The first is dissemination, which includes dissociation of cells from a primary tumour, migration to a nearby blood or lymph vessel, passage through the vessel wall and release into the blood stream (intravasation) [1], [9]. The second phase is colonization, which includes arrest of the cell at a particular point in the vessel system, penetration of the vessel wall by the cell, extravasation into the surrounding tissue (diapedesis) and establishment of secondary tumour sites when suitable biochemical and mechanical niches are available and angiogenesis supplies nutrients for sustained growth [1], [2].

Computational modeling has already played a notable role in cancer – in image registration and interpretation techniques, elastography and therapy design [29]–[33]. More recently, models of tumour growth and cell motility – including biochemically-motivated studies, Potts models and continuum models – have appeared [34]–[39].

The goal of the present study is to use a single, integrated, finite element-based model to investigate all of the steps in the dissemination process. Multiple means may exist to produce the requisite component motions, and this study investigates those that the authors deemed to be the most probable or dominant. In addition to providing information about the strength of the driving forces required, the model indicates the geometries cells would need to take in specific parts of the process, such as the various steps involved in traversal of a vessel wall. The study also aims to identify specific mechanical properties that pharmaceuticals or other interventions might target in order to thwart particular steps and thereby arrest metastasis.

## Mechanical Foundations of the Model

Fundamental to understanding the motions of cells is an understanding of how sub-cellular components can generate forces of the right kinds to produce these motions. The connections are not always apparent and sometimes they are counterintuitive. This fact alone suggests an important reason to use computational models: they are “ignorant” of the desired or observed result and so are unbiased. Motion of cells in aggregates is fundamentally different from that of cells that crawl on *in vivo* or *in vitro* substrates. In the latter case, a cell can use pseudopodia which attach to the essentially rigid substrate and by changing the pseudopod length or angle can move across it. Cells in aggregates do not have pseudopodia, but some have lamellipodia or contractile invadopodia that can attach to neighboring or next neighbouring cells and contract, thereby producing a certain degree of relative movement. Computational models have shown that the movements are relative and that the resulting global displacements are substantially less due to local recirculation [40]. Models have also shown that if the edges of aggregated cells contract and elongate in suitable ways, such as those consistent with the Differential Interfacial Tension Hypothesis (DITH), repeated actions can produce neighbour changes between cells and generate meaningful relative displacement of one cell relative to its neighbours and emergent behaviour such as sorting [21], [25]. The way this happens is not intuitive and computational simulations are necessary for testing hypotheses about the conditions needed to produce particular outcomes.

Now, consider the two cells labelled B and C in Fig. 2A that are in contact with each other. If the microfilament bundles shown schematically in red contract, they will tend to shorten the length of the boundary between **A** and B and will pull the triple junction between cells A, B and C downward (red arrows). These forces would be paired with corresponding upwards forces (equal and opposite, and for figure clarity not shown) at the lower end of the BC interface. Contractions in the cell membranes and their associated protein systems (together shown with black curves) will have similar mechanical effects. The mechanical result of these two systems operating together is a net contraction along the BC interface, denoted as an interfacial tension γ_BC_.

If adhesion systems are present, as suggested by the orange and blue dots along the membrane, they will also have a mechanical effect. In the figure, the adhesion molecules represented by the orange and blue dots fourth from the bottom of the figure are assumed to have just made binding contact, and the surrounding membrane is strained and will be so until the BC boundary elongates sufficiently at the ABC triple junction to relieve the strain. Inasmuch as the adhesion tends to elongate the edge (push the triple junction upwards as suggested by the blue arrow), it reduces the total contraction effect and lowers the net interfacial tension γ_BC_ [25], [41], [42].

Cells in mature tissues are typically held in place by extracellular matrix (ECM). In order for migrating metastatic cells to move, they must dissolve some of this matrix (represented by the orange extracellular curves in Fig. 2B), which they do using matrix metalloproteinases (MMPs) [43], [44]. Invadopodia are assumed to grow out from a host cell (cell E) as the ECM is suitably cleared away, attach to one or more neighboring cells (as suggested by the three short black lines to cell D) and then begin to contract. The resulting elevated tension along the DF interface can be modelled as an increased interfacial tension γ_DF_ [40], [45].

The steps outlined in this section indicate how a mechanics-based mathematical model is used to relate forces generated by various cytoskeletal components and other structural systems to cell-level characteristics, such as interfacial tensions. When multiple cells are in contact with each other, as in a typical tissue, these tensions interact with each other at each triple junction. Force equations could be written for each triple junction, but because the equations would be coupled together through cell volume constraints and the deformability of individual cells, the full set of motion equations would have to be solved simultaneously. One could not analyze the forces acting at a single triple junction and determine its motions independently from the motions of all of the other triple junctions. Setting up and solving the large sets of simultaneous equations that arise is not practical to do by hand, and instead one uses a computational model. When properly constructed, such models provide a consistent mathematical framework for setting up and solving the governing equations.

## The Computational Model

In this study, we use a 2D, cell-based computational engine [46] that was used extensively in the study of cell and tissue movement problems in the context of embryogenesis. This computational model is based on the widely-accepted finite-element (FE) method, a method that is used extensively in engineering and the physical sciences. The 2D version of the model was chosen over its 3D counterpart because studies based on the former are simpler to build, run, view and interpret. In addition, surface tension-based motions are governed by the same equations regardless of spatial dimension [47]. That being said, it is know that certain mechanics phenomena are dependent on dimension [48], and the authors look forward to the day when 3D simulations of metastatic processes can be carried out.

Other modeling approaches are possible, as well, including automata, lattice, Potts and centric models [21], [39], [49]. Features and drawbacks of each are covered in the cited articles. In brief, finite element models are more difficult to program and take longer to run than most of the other models, but the cell shapes they produce are based firmly on principles of physics, they offer realistic smooth cell edges, and the mechanical effects of the cytoplasm can be accounted for better than in many other methods. It should be noted that although the initial configurations used in the present model are Voronoi tessellations, it is a node-based model, not a centric one.

The FE model used here had previously been used to investigate the mechanics of cell and tissue annealing, dissociation, aggregation and engulfment [25], [50], [51]; cell sorting and mixing [21]; lamellipodium action and its role in convergent extension [45]; and tissue reshaping by directed mitosis [52]. Findings from these studies, incorporated into constitutive equations [53], made possible whole-embryo studies of neurulation [15], [17], the process through which the tube-like precursor of the spinal cord and brain forms. The model made it possible to trace the sequence of biochemical and mechanical events through which gene expression ultimately controls medical outcomes [17], [20].

A cell-based computational model of the kind used here calculates the forces generated in each cell, sets up equations to determine how those forces would interact with each other, and by solving those equations repeatedly determines how each cell will change shape and move over time [21], [42], [46], [51]. It can be used to answer questions such as, “Exactly what mechanical properties would a cancer cell on the surface of a tumour have to have (or acquire) so that it will dissociate itself from the tumour and move into the surrounding stroma?” It could also be used to address the question, “What properties would one have to change and by how much in order to sequester that cell and prevent it from leaving the primary tumour?” This mechanical information might then be used to figure out which sub-cellular structural components would have to be made more or less active in order to bring about those changes in properties. It might even be possible to determine which gene networks or receptors one might target using pharmaceutical or other means in order to bring about the necessary changes in those structural components.

In terms of the biology of the system, we assume that biochemical pathways (Fig. 3A) construct and regulate the operation of cytoskeletal components, adhesion mechanisms and other force-generating structures in the cell [17], [20]. These assumptions are identical to those used in common developmental biology simulations. Consistent with theoretical considerations, experiments and preceding explanations, we further assume that these structures generate net interfacial tensions γ (Fig. 3B) along the boundaries between cells and that the magnitude of these tensions is dependent on the types and states of the cells that form the interface [15], [25], [26], [41], [54], [55]. For example, if a migrating cancer cell (denoted as being of type C) is surrounded by normal tissue (N), the interfacial tensions along that boundary are denoted as γ_CN_ while the interfaces between normal cells are denoted as γ_NN_. In the present model, all homogeneous interfaces are given a tension of 10, while heterogeneous ones are given higher values. Some of these tensions change from one part of the metastatic process to the next, and values are reported in the corresponding figure captions, where appropriate, or in the text when longer explanations are required. Previous studies have shown that the patterns of behaviour that arise depend on the ratios of these tensions not their actual values [25]. Other designated cell types include tumour cells (T) and vessel wall cells (W), and the blood is denoted (B). The tensions that act along interfaces with the blood should strictly speaking be called surface tensions, but for grammatical simplicity they are referred to using the more generic term interfacial tensions. The values of γ_TW_, γ_TB_ and γ_NB_ are not assigned numerical values as the associated interfaces do not occur in these simulations. Many models of the mechanics of cells begin with assumed edge tensions or equivalent energy descriptions [42], [56] and doing so makes it possible to focus on the mechanical aspects of the situation. It also circumvents the problem that not enough is yet known about the gene networks and how they determine structural component forces that interfacial tensions could be determined quantitatively from them. This part of the formulation as well as the numerical implementation that follows are identical to those used in our previous developmental biology simulations.

The finite element model used here (Fig. 3C) is designed to match the geometries and mechanical properties of the individual cells involved (compare Figs. 3B and 3C). Interfacial tensions are implemented through rod-like elements along each cell-cell or cell-blood boundary [46], [51]. The rods have zero stiffness, but carry a constant axial force γ specific to the cell types that form the boundary. Multiple rods connect at each of the nodes and the net forces at those nodes are found by vectorially summing the forces from the individual rods. For organizational purposes, the vector sums at each node are then assembled into a matrix of nodal forces **f** [42], [57]. When lamellipodia or invadopodia are present (Fig. 3B and 3C), the rod along the interface where it acts is assumed to carry a tension that is q times the tension that would otherwise act along that edge [40]. In the present model, the process of invadopodium formation and extension is not explicitly modeled. The invadopodium is assumed to form instantaneously in its fully extended configuration at the beginning of a particular time step. Instead, the model focuses on invadopodium contraction and the cell movements and shape changes that it produces. The parameter q typically takes a value of approximately 2, a value consistent with the presence of 2 actual cell-cell boundaries between that pair of cells (e.g., cells D and F in Fig. 2B) rather than the usual 1.

The cytoplasm, intermediate filament networks and organelles inside the cells (Fig. 3A) are assumed to produce a net viscosity µ (Fig. 3C) [42], [53]. From a mechanical perspective, µ serves only to determine the rate at which the cells deform and move. For simplicity it is assigned a value of 1 in the calculations, but the exact value is inconsequential since all results are normalized. In the finite element model, the cell viscosity is represented by a system of orthogonal dashpots [46], one such system of which is shown in Fig. 3C. This approach avoids a stiffening artefact that arises when area triangles or some other standard approach is used to represent the cell volume. These dashpots are used to calculate a damping matrix **C** that is analogous to the stiffness matrix **K** used in elasticity problems. Details of the formulations are not given here as they have been presented elsewhere [46], [57]. The matrix equation
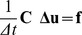
(1)is then solved for successive intervals of time Δt and the several hundred sets of incremental nodal displacements **Δu** it yields over the time course of the simulation give the detailed shapes and positions of each cell over time (as in Movie S1).

**Figure 4:**
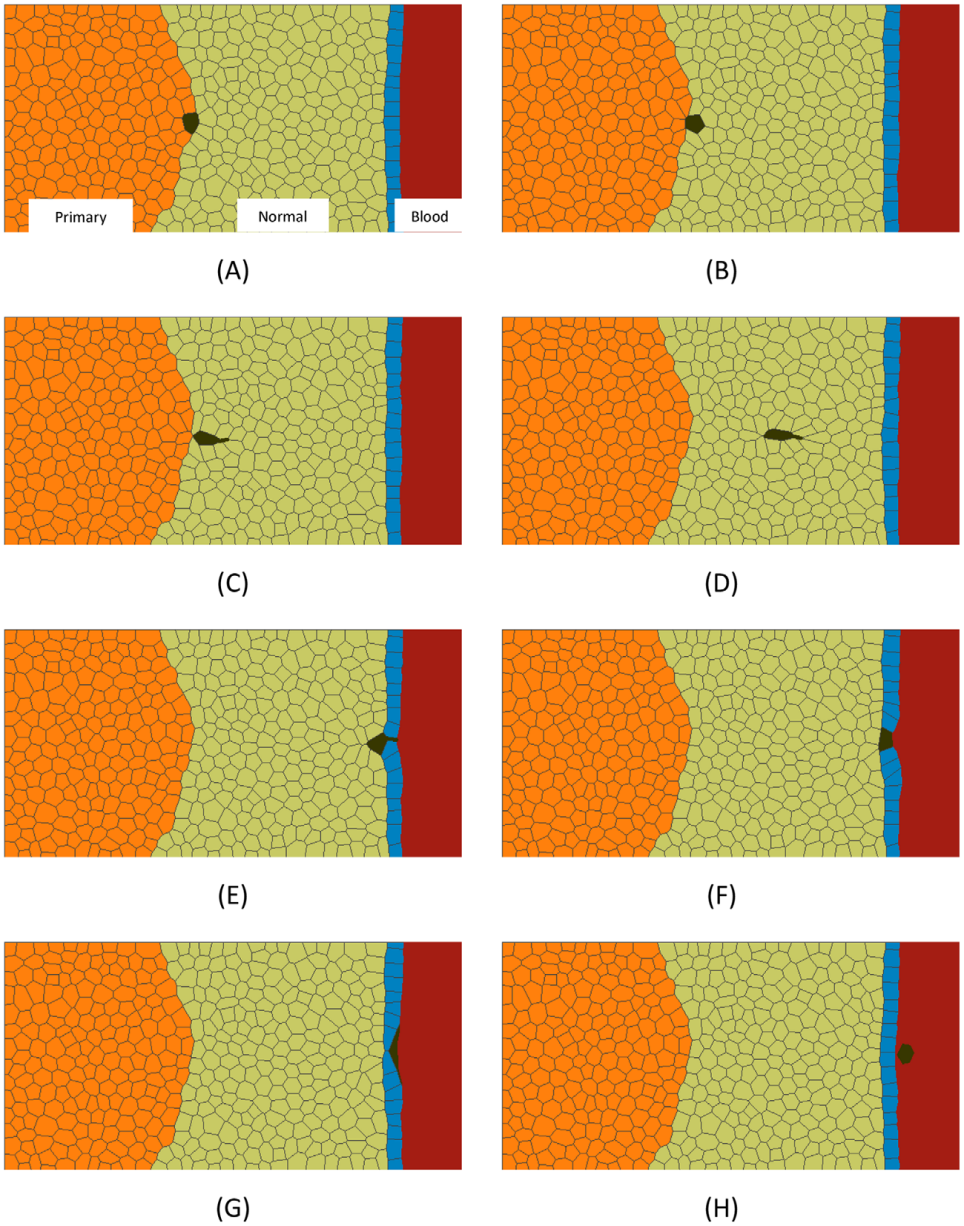
A simulation of the complete dissemination phase. All of the steps in the dissemination process are demonstrated in this simulation. In this simulation as in the others reported here unless noted otherwise, γ_NN_  =  γ_TT_  =  γ_WW_  = 10, γ_NT_  = 20, γ_NW_  =  γ_WB_  = 50, γ_CT_  = 40, γ_CN_  =  γ_CW_  = 20, q = 2. The starting configuration is shown in (A). The metastasis journey begins when a single cell in the tumour becomes sufficiently discriminated from its neighbours that Equation (2) is satisfied (see text). It is then pushed out of the tumour by interfacial tension differences (B) until at (C), its contact with the tumour becomes vanishingly small. Changes in cell signalling associated with loss of contact with the primary tumour or contact only with stromal cells or detection of chemotactic gradients are then assumed to initiate invadopodia that are oriented toward a nearby blood vessel and that pull the cell toward it (D). The migrating cell encounters the outside surface of the blood vessel (E) and continues to advance by invadopodium action until it makes contact with the blood stream (F). A further programming change in the cell occurs (γ_CN_ increases to 60) and it is pushed into the blood stream (G) and released there (γ_CW_ increases to 80) (H) by surface and interfacial tension differences akin to those that pushed the cell out of the primary tumour. See also Movie S1.

In a typical simulation, a starting geometry (Fig. 4A) consisting of several hundred cells of multiple cell types is constructed. The characteristics of each cell type are then defined in quantitative terms. These characteristics include the interfacial tension a cell of a particular type will generate along its edges when it is in contact with another cell of a specific type. They also include the mechanical specifications for any protrusions that that cell might generate. The software package then sets up the equations describing each cell, assembles them together into a large system of simultaneous linear equations and solves those equations to determine how the cells will move during the next short period of time. The equations are then updated to reflect the new geometries of the cells and any changes in their neighbours, and the equations are re-generated and re-solved (Fig. 4). These iterations continue until the single process or sequence of processes of interest to the user are complete. In practice, the user may estimate the number of time steps required in advance or may terminate the program based on a visual examination of live graphic output. The output of the model is a numerical description of how every node (Fig. 3C) of every cell moves with time, and drawings like Fig. 4 are simply graphical representations of the hundreds of numbers it generates for each time step.

The program acquires no useful information from the particular names assigned to the cells. Instead, the program begins with cell geometries as laid out in the initial configuration and based on specified interfacial tensions and other cellular properties it blindly sets up and solves the equations it constructs. As a result, its output is completely unbiased with respect to the supposed purpose of the cells it models or the motions the researcher hopes it will generate. Thus, the simulations can give unprejudiced answers to such questions as, “If an aggregate of cells with a specified initial geometry and with particular properties γ were allowed to interact with each other over time, what motions, if any, would result?

## Simulation Results

Figure 4 and Movie S1 show the full sequence of events through which a cancer cell leaves a primary tumour, migrates through stromal tissue, passes through the wall of a blood vessel and enters the blood stream. A single computational run was used to obtain all of the images, and the properties of the motile cancer cell were assumed to change in response to its current environment, as described in the figure caption. We now consider each part of the process.

### Dissociation

The first step a metastatic cell takes in its complex journey is to dissociate from a primary tumour. Experimental and computational studies of embryonic tissues [25], [41], [55], [58]–[60] have shown that aggregation, engulfment and dissociation of cells and tissues are driven by differences in interfacial (and surface) tensions. These tension differences are a necessary condition for cell motions to occur.

One of the most distinctive identifying marks of cancer cells is the changes that occur in their surface properties, including their surface markers, surface tensions and integrin structure and activity [1], [9], [28], [61]. This suite of changes is consistent with that needed to produce motility, including dissociation from a primary tumour. Previous studies have shown that changes in the tension in the cell membrane, degree of activity of actomyosin and other cortical contraction systems, and surface adhesion systems all affect net interfacial tensions [25], [41], thus providing possible mechanisms for the changes in net surface tension needed to produce cell dissociation.

Metastases occur in mature tissue, where ECM tends to lock cells into position, unlike in embryonic tissues where less extracellular structure is present and cells are relatively mobile. The same mechanical force principles govern both tissues, but for cell movements to occur in mature tissue the ECM must yield to physical or chemical actions. That cancer cells do become mobile demonstrates that this occurs, and MMPs have been identified as a primary means for ECM remodeling, and their association with tumour cell dissemination has been recognized [43], [44].

**Figure 5:**
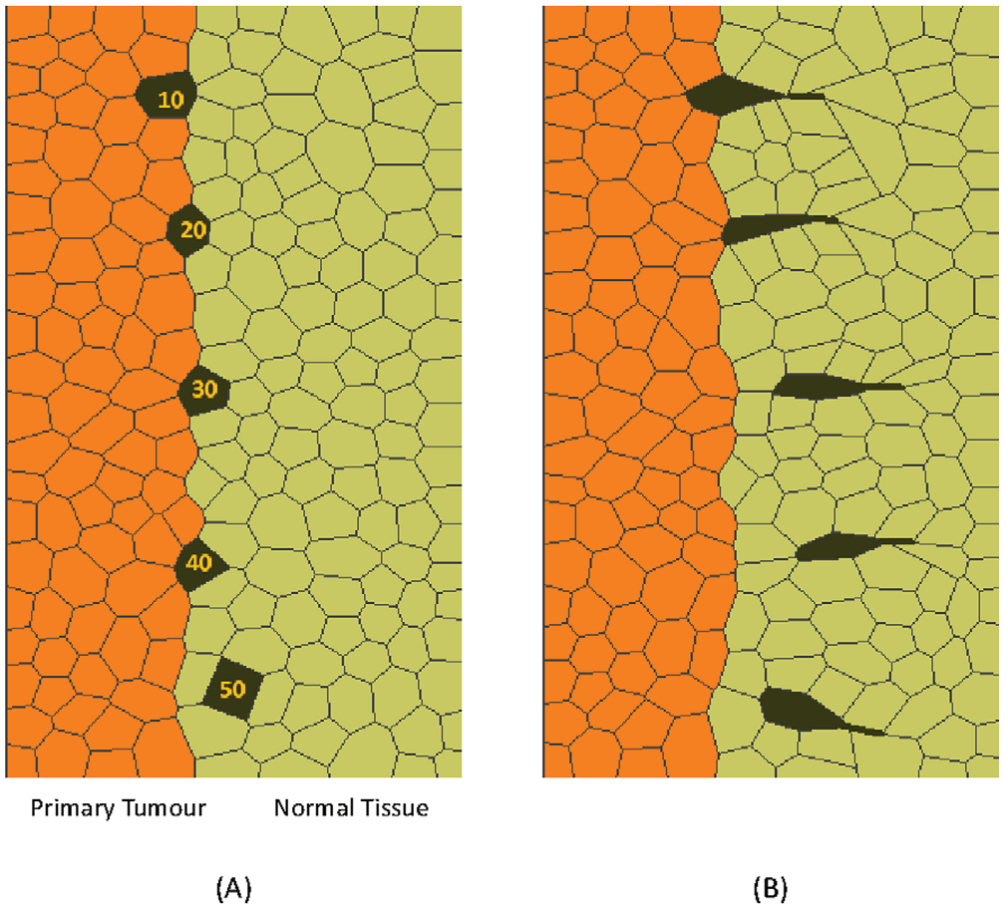
Simulations of dissociation. Part (A) shows a series of simulations in which the strength of the tension along the cancer cell-tumour boundary takes on a variety of values from γ_CT_ = 10 to 50. The case with γ_CT_ = 50 is the only one that satisfies Equation (2) and it is the only one that successfully escapes the tumour. In (B), the activated cells are assisted by invadopodia and cells with γ_CT_ as low as 30 are able to escape.



Previous cell-level simulations of cells, physics-based theoretical analysis and experiments [25], [41], [62] have shown that for a single cell to leave a mass, the boundary between that cell and the mass must shorten to zero length. In order to do so, it must typically elongate the boundaries between that cell and the material surrounding the mass (eg., the surrounding normal cells or, if in contact with liquid, the surrounding medium), and the boundary between the mass and the surrounding material. Figure 5A shows how the length of the tumour-cancer cell boundary shortens as γ_CT_ increases until it shortens to zero length and the cancer cell becomes freed from the tumour. The various changes in boundary lengths alter the free energy of the system and for the cell to leave the mass, the following equation, written in terms of the cell types used here, must be satisfied:

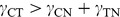
(2)The equation can equally well be understood in terms of the forces acting at the triple junction between the C, T and N cells: The force along the boundary between the dissociating cell and the rest of the tumour must be sufficiently high that it can overcome the resisting forces produced by the tensions along the dissociating cell-normal boundary and tumour-normal cell boundary. If it is sufficiently strong, it can shorten the CT boundary to zero length, at which point the cell C becomes dissociated from the tumour mass (Figs. 4 and 5A). If these force conditions are not met, the cell cannot leave the primary tumour regardless of whether the ECM is remodelled in ways to accommodate such motion or not. The simulations shown in Fig. 5A illustrates how changes to the value of γ_CT_ would affect the cell shapes and degree of expulsion produced. Figures of this type may be useful in evaluation of the forces at work in cells shown in histological sections and other experimental data. Future simulations could model the EMC explicitly [34], [49] as equations describing its constitutive equations and distinctive characteristics become known, but such enhancements would not change the basic requirement embodied in Eqn (2).

For a single cell to leave a primary tumour, its mechanical properties must differ in a suitable manner from those of its immediate neighbours as described above. If all cells in a tumour had the same mechanical properties either they would all stay together in a compact mass or they would all dissociate from each other [25]. Modern techniques have shown that heterogeneity of cell type is another of the distinguishing features of cancerous tumours [5]. Thus the force differential equation (Eqn 2) must be satisfied only for a single surface cell and be satisfied only long enough for that cell to be released. Cancers can involve the flow of multiple cells away from a primary tumour [1], but the mechanics of those cases are somewhat more complex and beyond the scope of this study.

Equation (2) is not sufficiently general to account for situations where a cellular protrusion acts on a cell that is still part of a primary tumour, and to address this circumstance requires simulations like those shown in Fig. 5B. Those simulations demonstrate that protrusions can make a difference in whether or not a cell leaves a primary mass. However, the protrusions were not found to generate sufficient force by themselves to pull a single cell away from a primary tumour. Whether joint action might occur *in vivo* has evidently not yet been investigated, but simulations can still investigate the concept and reveal the cell shapes and other geometric features one would expect to find if they did act. Indeed, use of simulations in this way has become increasingly common in developmental biology [16], [40], [63] and it illustrates the healthy dialogue that can exist between experiments and models.

In terms of preventing metastasis, the obvious and preferred strategy would be to thwart dissociation, because then all potentially active tissue would remain localized and could be resected surgically. If one or more of the interfacial tensions is changed so that equation (2) is not satisfied then, assuming that protrusions are not active, the cancer cell cannot leave the primary tumour (Fig. 5A). If one were to introduce pharmacological or other interventions, one would presumably selectively target the cancer cells, and one might focus on the CT interface, where reducing the tension there by enough that equation (2) is not satisfied would be sufficient to prevent the metastatic process from getting started. A variety of structural components including those in the actomyosin and adhesions systems are involved in generating these forces, and gene networks, signalling pathways or metabolic factors that reduce γ_CT_ by increasing the adhesions at this boundary or reducing the cortical contractions might be targeted. Another approach would be to selectively increase the tension on the TN interface, and this might be achieved by reducing TN adhesions or increasing the cortical tension along this boundary.

From a mechanical perspective, the conditions needed for a cell to dissociate from a tumour (Eqn 2) are remarkably similar to those required for neural crest migration and other events during early embryo development [64], [65] – yet another cancer-embrogenesis connection. Both events are considered a form of epithelial-mesenchymal transition (EMT) and both require the interfacial tensions of the dissociating cell to change such that a cell that was homotypic with its neighbors now dissociates from them and enters an adjacent tissue [64]–[68]. A number of anti-metastatic pharmaceuticals in the pre-clinical stage target exactly this transition with the goal of preventing metastasis at the cell dissociation stage [69].

### Migration

The next step in the metastatic journey involves migration of a dissociated cell to a nearby blood or lymph vessel (Fig. 4D). In metastatic cells, this process evidently happens through the repeated extension and contraction of a lamellipodium-like protrusion called an invadopodium [1], [9], [70]. Fortunately, the computational model used to study dissociation had been extended previously so that it included lamellipodia and other protrusions and had been tested extensively in the context of lamellipodium-driven convergent extension [15], [40].

In the present model, when a cell dissociates from a primary tumour, an invadopodium is assumed to grow out from it and to push its way through the cell-cell boundary that is pointed most directly toward the vessel wall (Figs. 2B, 3A, 4C and 4D). Clearly this change in activity requires that specific gene circuits and feedback mechanisms be activated in the cell and that chemotactic gradients or other directional cues be provided via the stroma in which it resides [1], [8], [71]. How these directional cues are created and the pathways through which they function are important considerations. However, these circuits are not explicitly incorporated into the present model, but rather this model used a high-level surrogate function that assumes the required migration direction is known and that invadopodia form as required to drive cell motion. This is a reasonable step in a model whose goal is to elucidate the mechanical aspects of the process. In time, one hopes that models can explicitly model the generation of chemotactic gradients, the mechanisms that cells use to detect them and the molecular pathways by which specific cytoskeletal components are constructed and activated. For similar reasons, the process of ECM remodeling is not explicitly modeled and, as a consequence, the current model cannot be used to draw conclusions about the time rate of cell movement. In these simulations, the invadopodium extends until it contacts the next neighbouring cell, at which point it is assumed to generate a tension that is q times that of the tensions along the cell-cell boundaries in the surrounding area. When q is equal to 2, it produces motions and cell shapes consistent with those in histological sections. For comparison purposes, simulations were run using higher and lower q values, and these showed that when q is less than approximately 2, the invadopodium does not contract significantly and the dissociated cell does not migrate. If it is made larger than approximately 2.5, the invadopodium contracts rapidly and elongates the dissociated cell considerably, but as soon as the invadopodium shortens to zero length and stops pulling, the cell rapidly returns back to its previous shape, and useful motion is not produced. However, when q = 2, reliable motion and reasonable cell shapes result.

From a practical point of view, preventing migration is nearly as good a preventing dissociation because in both cases all of the cancer cells are tightly grouped together and can be surgically removed from a single site. In terms of preventing migration, one might consider strategies that interfere with invadopodium extension, attachment, or contraction. The simulations suggest that if the relative contraction force q were either smaller than about 2 or greater than 2.5 migration could be prevented. From a medical point of view, it is good that the range of values needed for migration is relatively small, suggesting that interventions that target this value might be successful. The groundwork is still being laid for the development of pharmaceuticals that target this phase of the metastatic process [72].

### Intravasation (and Release)

Passage through the vessel wall is a complex, sequential process and for simplicity, the present model assumes the blood (or lymph) vessel wall to consist of a monolayer, rather than a multi-layer structure as is more common *in vivo*. This simplification does not reduce the validity of the simulations, because the same kinds of driving forces would be necessary regardless of the number of layers. If a multi-layer geometry were present, the governing equations would have to be satisfied in sequence as the cell enters, traverses and exits each layer. In a mono-layer configuration, the steps and their associated equations need be considered only once.

**Figure 6:**
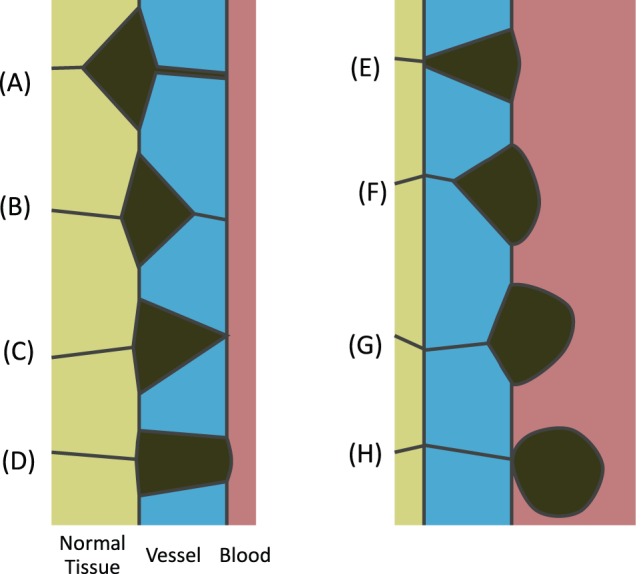
The process of intravasation. When a migrating cell arrives at a blood vessel, invadopodia of suitable strength (A) or surface tension differences (B) can pull it into the vessel and bring it in contact with the blood stream (C). Further details are given in the text. At that point, invadopodia can no longer pull the cell forward and interfacial tensions must drive further advancement (D–F). Eventually, the cell is in contact only with the inner layer of the blood vessel and if γ_CW_ is sufficiently strong that Equation (3) is satisfied, the CW boundary will shorten (G) to zero length and the cancer cell will be released into the blood stream (H).

Figure 6 illustrates a cancer cell (shown in dark green) leaving normal or stroma cells (light green), traversing a vessel wall (blue) and entering the blood (reddish grey). In part A of the figure, ingression of the cancer cell into the vessel is driven by an invadopodium (as in Fig. 4 and Movie S1), while in B it is driven by interfacial tension differences (γ_WW_ is sufficiently greater than γ_CW_ that the triple junction is moved across the thickness of the vessel). In either case, the configuration shown in Fig. 6C results. The q and interfacial tensions reported in the caption of Fig. 4 are one combination of values that achieve the sequence of events shown in Fig. 6. Other values could work, and the limits of those values would depend on the thickness of the vessel wall and other geometric and mechanical parameters, and could be determined through further sets of simulations. This essentially completes leaving of the cell from the stroma and its ingression into the vessel.

Next the cell traverses the vessel, and this involves at least two aspects. For the invading cell to have more than point contact with the blood stream the adjoining cells must retreat away from it. If this is not true, the cancer cell will not be able to open a hole in the vessel through which to exit. Specifically, the cancer cell-blood interface must expand as in Fig. 6D (γ_CB_ must not be too large compared to the other cancer cell tensions and γ_WB_) and the cancer cell-normal interface must contract to zero length as in 6E (γ_CN_ must be sufficiently large compared to the other cancer cell tensions and γ_NW_). Signals from the blood or normal cells may cause the tensions to change as the cell forms contacts with the blood and loses its contact with the normal cells.

Finally, the cancer call must be expelled from the vessel wall (Figs. 6F through H), or conversely drawn into the blood stream. This process is mechanically different from that by which it breaches the vessel wall. Although anatomists might consider traverse of the wall to be a single event, mechanically, it consists of two distinct phases. The first part of it requires that γ_WW_ not be too large compared to γ_ CW_ so that the triple junction can move across the vessel wall. Then, in a process parallel to that by which the cancer cell initially pinched off from the primary tumour, it must pinch off the vessel wall. This last step requires.
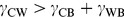
(3)


For a cell to traverse a multi-layer vessel, these steps would have to be repeated for each layer, and the properties of the cancer cell may need to be changed in response to the cells with which it is currently in contact. In time, cell signalling details will be worked out, and they could then be incorporated into finite element or other types of models [35], [37] and used to gain a systems understanding of the intravasation process.

Changes to the invadopodium force (i.e., to q) or interfacial tensions would be sufficient to block the initial intravasation step (Fig. 6A), but would seem a poor clinical strategy since it would cause cancer cells to collect and form a layer around the outside of the vessel. Similarly, blocking the release step by ensuring that equation (3) is not satisfied would cause cells to collect on the inside of the vessel leading to stenosis or occlusion if they remained in place, or possibly to stroke if they broke off in aggregates, neither of which is an acceptable clinical solution. On the other hand, if the migrant cancer cells have impinged only vessels within one to two mm of the surface of the primary tumour, resection of the tumour and immediately surrounding vasculature might still be an acceptable surgical strategy.

### Circulation

As long as Eqn (3) remains true for all vessel tissues that the circulating cancer cell might touch, it will not reattach. This observation suggests that single cells along the vessel wall whose properties even temporarily violate Eqn (3) might be sufficient to arrest cell circulation and initiate colonization.

## Discussion and Conclusions

The model presented here provides a new, mechanical vantage point for considering the process of cancer metastasis. Specifically, it provides a new framework for posing and testing hypotheses about the mechanical forces that drive each of the steps involved in the spread of cancer. The model shows that regardless of the state or action of any genetic or signalling networks, or of any proteins or other molecules in the cell, if mechanical forces of the right magnitude and type are not ultimately generated, cancer cannot spread. Thus, one might, as embryologists have increasingly done, use mechanics as the starting point and trace cause and effect backwards to cytoskeletal and other force-generating structures and, when possible, backward from there to the biochemical networks that govern them.

Although the geometry of the model used here is considerably simplified compared from that of real tissues and the number of cells is much smaller than would be found *in situ*, these simplifications do not change the basic character of the forces needed to drive the motions of interest. Instead, they make it easier to identify the key parameters and focus on the essential mechanical characteristics and phenomena. The model presented here, evidently the first finite element model of metastasis, is offered in the first instance as a proof of principle. In time, one could build models based on measured mechanical properties of specific cells and tissues, and one might include signalling and regulation pathways for structures that generate these forces [35], [37], [49]. The models might also be carried out in three-dimensions, which may reveal some differences compared to 2D models [48].

The model unexpectedly led us to recognize the possible importance of single cell properties and temporary states. For example, dissociation requires a potentially migrant cell to become mechanically different from its neighbours in a specific way (Eqn 1) and to do so only for long enough that the cell can escape the tensions that would otherwise hold it in place. Future models might consider the consequences of stochastic variations in all of the cells near the tumour surface and might produce a probability distribution for cell release. This is one of many ways in which computational models can provide a richer understanding of a biomechanical event. The migration, intravasation, release and arrest steps all involve mechanical events that could be moved past a tipping point by stochastic events at the mechanical, ultra-structural or biochemical levels, and would be worth modeling in future. The models needed could, presumably, be enhancements of the one used in the present study. Measurements of cell properties (“omic”, morphological and mechanical) and their changes over time would be of great value in constructing and validating such models.

The literature is full of intriguing information about mechanical factors associated with cancer, and it is natural to ask whether mechanical models can provide insights into them. Some of the questions one might consider include:

1) Is the increased stiffness of cancer tissue [31], [73] related to the altered surface tensions in the cells of which it is composed and elevated resulting intracellular pressures? Can these mechanical properties be related mathematically to each other?
2) Why does mechanical stimulation increase the rate of cell invasion [10]? Can the mechanotransduction feedback loop involving cofilin be better understood with the aid of mechanical models?
3) Do the mechanical properties of cells near the primary tumour affect dissociation or is the effect of nearby cells on invasion exclusively biochemical [7], [10], [74]?
4) Can stochastic models be used to characterize the dissociation process? Can such models identify specific types of cells that while they do not metastasize themselves cause other cells to do so? Can probability distributions be constructed for dissociation and the other steps in metastasis?
5) What can models tell us about the leading edge of invadopodia and the properties they need?
6) If the rate constants associated with ECM remodelling are incorporated, can the model predict the stochastic rates of cell motion.
7) Can the intravasation process be modeled in more detail so that the physical structure of the various layers of the vessel and their distinctive mechanical properties can be taken into account?
8) Can the mechanical properties of cells be inferred from their geometries or motions? A new model-based technique called Video Force Microscopy (VFM) has been able to determine the forces acting in moving embryonic cells [18]. Can a similar approach be taken to the study of cancer cells?
9) What can mechanical models tell us about the various steps in the colonization phase of metastasis?
10) Can the models explain how stress or other temporary or long-term systemic factors affect the incidence of metastases?
11) Can models help us to understand why cells of particular cancer types preferentially colonize tissues of specific other types?

There is no technical reason that such questions could not be answered using suitably-constructed computational models. However, to address questions of this kind will require dialogue and collaboration between experimentalists and modellers. Models with additional mechanical and biochemical features will have to be built, new experiments carried out and old ones re-examined in light of the models, and the models revised to incorporate the new experimental findings. When intense interdisciplinary interactions of this nature began to occur in the field of embryology, many important new discoveries were made. Thus, there is good reason to believe that collaborations between modellers and experimentalists will lead to equally significant advances in cancer prevention.

## Supporting Information

Movie S1 **A simulation of the process of cancer metastasis (dissemination phase).**
(MPG)
